# Mapping Health Disparities in 11 High-Income Nations

**DOI:** 10.1001/jamanetworkopen.2023.22310

**Published:** 2023-07-07

**Authors:** Neil J. MacKinnon, Vanessa Emery, Jennifer Waller, Brittany Ange, Preshit Ambade, Munira Gunja, Emma Watson

**Affiliations:** 1 Department of Population Health Sciences, Augusta University, Augusta, Georgia; 2 Office of the Provost and Institute of Public and Preventive Health, Augusta University, Augusta, Georgia; 3 International Program in Health Policy and Practice Innovations, Commonwealth Fund, New York, New York; 4 National Health Service Education for Scotland, Edinburgh, Scotland

## Abstract

**Importance:**

Health care delivery faces a myriad of challenges globally with well-documented health inequities based on geographic location. Yet, researchers and policy makers have a limited understanding of the frequency of geographic health disparities.

**Objective:**

To describe geographic health disparities in 11 high-income countries.

**Design, Setting, and Participants:**

In this survey study, we analyzed results from the 2020 Commonwealth Fund International Health Policy (IHP) Survey—a nationally representative, self-reported, and cross-sectional survey of adults from Australia, Canada, France, Germany, the Netherlands, New Zealand, Norway, Sweden, Switzerland, the UK, and the US. Eligible adults older than age 18 years were included by random sampling. Survey data were compared for the association of area type (rural or urban) with 10 health indicators across 3 domains: health status and socioeconomic risk factors, affordability of care, and access to care. Logistic regression was used to determine the associations between countries with area type for each factor, controlling for individuals’ age and sex.

**Main Outcomes and Measures:**

The main outcomes were geographic health disparities as measured by differences in respondents living in urban and rural settings in 10 health indicators across 3 domains.

**Results:**

There were 22 402 survey respondents (12 804 female [57.2%]), with a 14% to 49% response rate depending on the country. Across the 11 countries and 10 health indicators and 3 domains (health status and socioeconomic risk factors, affordability of care, access to care), there were 21 occurrences of geographic health disparities; 13 of those in which rural residence was a protective factor and 8 of those where rural residence was a risk factor. The mean (SD) number of geographic health disparities in the countries was 1.9 (1.7). The US had statistically significant geographic health disparities in 5 of 10 indicators, the most of any country, while Canada, Norway, and the Netherlands had no statistically significant geographic health disparities. The indicators with the most occurrences of geographic health disparities were in the access to care domain.

**Conclusions and Relevance:**

In this survey study of 11 high-income nations, health disparities across 10 indicators were identified. Differences in number of disparities reported by country suggest that health policy and decision makers in the US should look to Canada, Norway, and the Netherlands to improve geographic-based health equity.

## Key Points

**Question:**

Are there geographic health disparities across 11 high-income countries?

**Findings:**

In this survey study using self-reported data from 22 402 participants across 11 countries, the mean number of geographic health disparities across 10 indicators and 3 domains (health status and socioeconomic risk factors, affordability of care, access to care) was 1.9, although there was wide variation among the nations. The US had significant geographic health disparities in 5 indicators, the most of any country, while Canada, Norway, and the Netherlands had no significant geographic health disparities.

**Meaning:**

These results suggest that health policy makers in the US should look to Canada, Norway, and the Netherlands to improve geographic-based health equity.

## Teaser

This survey study compares geographic health disparities in socioeconomic risk factors, affordability of care, and access to care across 11 high-income countries.

## Introduction

Geographic health disparities are well recognized in public health and health care delivery. The health of people in rural areas in particular is a recognized concern in the US and globally.^1,2,3^ In the US, the death rate is higher for rural vs urban areas (831 per 100 000 vs 704 per 100 000).^4^ Rural residents in the US have higher rates of chronic diseases^5,6^ (diabetes, obesity, chronic obstructive pulmonary disease), stroke,^5,6^ unintentional injury,^5,6^ tobacco use,^7^ opioid overdose,^8^ cancers associated with modifiable risks,^9^ suicide,^10^ worse maternal health,^11^ decreased confidence in the health care system,^12^ and limited access to care.^13,14,15,16,17^ In 2019, mortality rates were higher for the 10 leading causes of death in rural areas.^18^ Geographic health disparities also occur in other high-income countries, with evidence of overall worse rural health in Australia^19^ and Canada.^20,21,22^ In France and the Netherlands, there is evidence of worse perinatal health outcomes for women who live farther from hospitals, indicating that living in a rural area is a risk factor.^23^

Within the US and globally, the COVID-19 pandemic brought the geographic health disparities of rural areas to the forefront and exacerbated them. Rural populations had higher death rates from COVID-19.^24^ The Omicron variant hit rural areas of the US harder, where the vaccination rates were lower.^25,26^ Furthermore, practitioner shortages and facility closures accelerated during COVID-19, building on pre-COVID-19 trends.^27,28^ While the US invested more resources toward rural health during this time,^29,30^ further interventions are needed to promote geographic health equity in the US and abroad.

As these examples make clear, geographic inequities exist, but researchers have limited understanding of how to compare disparities across the globe. The Commonwealth Fund’s 2020 International Health Policy (IHP) Survey is one of the few surveys that collects data across these countries that is not specific to 1 disease or health condition. A study describing national-level differences is already published.^31^ Using the same data set, we conducted a secondary analysis to compare differences in geographic-based (rural vs urban) health inequities related to socioeconomic and health status, affordability of care, and access to care. This study explores geographic health disparities in high-income countries, with an emphasis on the US.

## Methods

We conducted a secondary analysis using data from the 2020 IHP survey. The survey collected data from a representative sample of adults ages 18 years and older across 11 high-income countries: Australia, Canada, France, Germany, New Zealand, Norway, Sweden, Switzerland, UK, US, and the Netherlands. It included questions about 10 different indicators across 3 domains: health status and socioeconomic risk, affordability of care, and access to care. A detailed methodology of the survey has been published elsewhere.^31^ Each country included in the survey defined rurality differently. We used these definitions to construct our main projection variable. A full description of the country-specific definition of rurality is listed in eTable 1 in Supplement 1. The Augusta University institutional review board determined that the study was exempt from human participant review and informed consent requirements waived because data were deidentified. We followed the American Association for Public Opinion Research (AAPOR) reporting guideline for survey studies and have provided details about the sample and sampling methods, range of margins of error, analyses we used, and citations to additional information about the survey and sampling methodology, as well as to the full questionnaire.

### Sampling Methodology and Weighting

The 2020 IHP Survey is conducted within each of the 11 countries to produce a nationally representative sample of individuals using probability sampling. The margins of error in sampling for each country ranged between 2.2 to 5.0. Details of the sampling methodology are available elsewhere.^31^ The full survey questionnaire is available from the Commonwealth Fund.^32^ A complex sampling design was used within each country, with each country using a different stratum to sample from. Random digit dialing (RDD) was used in Australia, Canada, France, Germany, the Netherlands, New Zealand, and the UK; the US used a hybrid addressed-based sampling frame or RDD; Norway used simple random sampling; and Sweden and Switzerland used population-based registries for sampling. Weights were determined within the stratification variables for each country based on the sampling design, probability of selection, and systematic nonresponse across known population parameters. The weights across all individuals within each country summed up to the number of individuals sampled within each country.

### Statistical Analysis

All statistical analyses were performed using SAS version 9.4 (SAS Institute Inc) and statistical significance was assessed using an α level of .05. All statistical analysis incorporated the complex sampling design within each country by incorporating the stratification and weights. Weighted percentages were determined within the country for demographic, health status and socioeconomic risk, affordability of care, and access to primary care indicators. Likewise, the percentage of rural residents within each country and health status and socioeconomic risk, affordability of care, and access to primary care indicator were determined.

Logistic regression was performed to determine the associations between health status and socioeconomic risk factors, affordability of care, and access to primary care (dependent variables) with the type of area (rural or urban) between countries (projection variable). We included age group, sex, and country as additional covariates. The interaction between country and type of area was included to determine whether the association between the health disparities and type of area differed between countries. Odds ratios (OR) and Wald-based 95% CIs were calculated for the type of area (rural vs urban) for each country.

## Results

The sample included adults 18 years of age and older across 11 high-income countries: Australia, Canada, France, Germany, New Zealand, Norway, Sweden, Switzerland, UK, US, and the Netherlands. The total sample size across all 11 countries utilized in the analysis was 22 402 (12 804 female [57.2%]), which represented a 14% to 49% response rate, depending on the country. The weighted descriptive statistics within each country for demographics, health status and socioeconomic risk, affordability of care, and access to primary care indicators are shown in Table 1. Respondents living in rural populations ranged between 84 of 753 (11.2%) in the Netherlands and 421 of 1003 (42.0%) in New Zealand. The percentage of male and female respondents was roughly equal and there was representation across all age groups.

**Table 1.** Descriptive Statistics for Demographic, Health Status and Socioeconomic Risk, Affordability of Care, and Access to Primary Care Factors in 11 Countries

Variable or factor	Respondents, No. (%) (N = 22 402)										
	Australia (n = 2201)	Canada (n = 4530)	France (n = 3028)	Germany (n = 1004)	Netherlands (n = 753)	New Zealand (n = 1003)	Norway (n = 607)	Sweden (n = 2513)	Switzerland (n = 2284)	UK (n = 1991)	US (n = 2489)
Demographic characteristic											
Rurality	679 (30.5)	894 (16.0)	976 (34.2)	285 (27.9)	84 (11.8)	421(45.0)	186 (34.3)	712 (27.7)	561 (27.3)	616 (22.7)	496 (18.4)
Sex											
Female	1192 (50.9)	2859 (51.1)	1838 (53.0)	572 (51.5)	384 (50.5)	580 (51.9)	311 (49.0)	1319 (50.0)	1167 (51.2)	1088 (51.3)	1494 (52.6)
Male	1009 (49.1)	2212 (48.8)	1190 (47.0)	429 (48.5)	369 (49.5)	423 (48.1)	296 (51.0)	1194 (50.0)	1114 (48.8)	903 (48.7)	983 (47.2)
Other	0	3 (0.1)	0	0	0	0	0	0	0	0	9 (0.3)
Age, y											
18-24	165 (11.9)	331 (11.2)	212 (10.2)	53 (8.4)	39 (9.2)	89 (12.3)	35 (10.2)	197 (10.2)	139 (8.9)	163 (11.0)	237 (10.8)
25-34	338 (19.4)	648 (16.7)	446 (15.4)	96 (15.3)	81 (15.6)	185 (18.7)	61 (16.3)	242 (16.5)	313 (16.4)	269 (17.2)	539 (17.8)
35-49	429 (25.0)	1204 (24.9)	692 (24.3)	213 (22.7)	149 (24.5)	237 (24.9)	138 (26.2)	512 (24.1)	616 (25.9)	407 (24.7)	548 (25.0)
50-64	506 (22.9)	1432 (27.3)	890 (24.8)	344 (27.6)	237 (26.5)	227 (24.5)	163 (24.6)	646 (23.4)	678 (25.9)	531 (23.9)	588 (26.2)
≥65	742 (20.8)	1395 (19.9)	779 (25.4)	297 (25.9)	247 (24.1)	258 (19.6)	210 (22.7)	916 (25.8)	537 (22.8)	600 (23.2)	574 (20.22)
Health status and socioeconomic risk factors											
Multiple chronic conditions	596 (21.3)	1143 (21.3)	336 (11.5)	178 (15.8)	107 (11.7)	173 (16.6)	121 (19.4)	613 (21.5)	331 (14.0)	410 (19.2)	704 (28.4)
Mental health condition	543 (27.5)	1125 (26.6)	451 (15.2)	114 (10.3)	102 (15.2)	181 (20.2)	97 (19.8)	410 (18.5)	325 (15.3)	467 (26.3)	781 (29.0)
Experienced material hardship	192 (9.1)	463 (12.7)	325 (13.1)	62 (6.1)	30 (5.2)	69 (8.7)	12 (2.9)	130 (6.3)	349 (16.4)	149 (10.9)	415 (16.3)
Affordability of care											
Skipped needed medical care	395 (21.2)	606 (14.4)	313 (11.1)	100 (10.5)	56 (9.4)	148 (17.8)	30 (8.2)	229 (11.2)	504 (25.4)	125 (9.6)	979 (39.0)
Skipped dental care	605 (31.7)	1120 (27.2)	553 (18.5)	174 (19.2)	67 (10.3)	346 (37.1)	86 (21.4)	479 (22.2)	606 (26.5)	293 (20.7)	923 (36.2)
Serious problems or unable to pay medical bills	161 (9.1)	271 (7.1)	262 (10.1)	40 (4.1)	30 (5.4)	53 (7.8)	23 (6.3)	157 (7.5)	188 (9.0)	52 (3.9)	542 (22.4)
Access to primary care											
Regular physician or place of care	2073 (93.0)	4698(89.9)	2880 (95.4)	973 (95.9)	748 (99.2)	961 (96.3)	607 (100.0)	2227 (86.7)	2117(93.0)	1951 (96.8)	2215 (89.2)
Same day care needed last time	1402 (65.0)	1786 (38.2)	1468(53.3)	705 (74.6)	445 (66.3)	598(60.5)	257 (46.9)	742 (33.0)	1096 (53.4)	991 (51.7)	1047 (49.2)
After-hours care difficult to access	868 (44.4)	2697 (60.0)	1238 (56.7)	359 (53.1)	124 (28.0)	314 (44.1)	145 (35.5)	898 (76.3)	774 (60.1)	892 (63.3)	1091 (54.1)
Avoidable ED visit past 2 y	175 (30.3)	753 (38.8)	207 (24.7)	68 (28.2)	41 (31.4)	61 (26.2)	37 (28.0)	207 (27.9)	220 (36.1)	185 (31.3)	306 (38.7)

Abbreviation: ED, emergency department.

The weighted descriptive statistics for each health status and socioeconomic risk, affordability of care, and access to primary care indicator among rural participants by country are given in Table 2. The country with the lowest proportions of respondents having multiple chronic conditions (178 of 1004 [15.8%]), mental health conditions (114 of 1004 [10.3%]), or experiences of material hardship (62 of 1004 [6.1%]) was Germany. The US had the highest, or one of the highest, rates for having multiple chronic conditions (704 of 2489 [28.4%]), mental health conditions (781 of 2489 [29.0%]), or experiencing material hardship (415 of 2489 [16.3%]).

**Table 2.** Percentage of Health Status and Socioeconomic Risk, Affordability of Care, and Access to Primary Care Factors Among Rural Participants in 11 Countries

Factor	Rural participants, No. (%)										
	Australia (n = 679)	Canada (n = 890)	France (n = 976)	Germany (n = 283)	Netherlands (n = 84)	New Zealand (n = 421)	Norway (n = 186)	Sweden (n = 712)	Switzerland (n = 560)	UK (n = 616)	US (n = 495)
Demographics											
Sex											
Male	288 (47.8)	360 (46.5)	362 (44.4)	123 (48.2)	41 (54.2)	164 (43.7)	96 (48.4)	350 (50.4)	275 (52.4)	274 (46.1)	181 43.5)
Female	391 (52.3)	530 (53.5)	614 (55.6)	160 (51.8)	43 (45.8)	257 (56.3)	90 (51.7)	362 (49.6)	285 (47.6)	342 (53.9)	314 (56.6)
Other	0	0	0	0	0	0	0	0	0	0	0
Age											
18-24 y	28 (8.8)	21 (3.8)	58 (8.0)	8 (5.0)	6 (11.5)	39 (14.0)	10 (7.5)	31 (6.1)	45 (12.2)	35 (6.3)	35 (6.4)
25-34 y	40 (8.2)	67 (11.6)	119 (13.6)	22 (13.0)	18 (13.6)	51 (10.9)	9 (8.9)	48 (13.1)	77 (15.6)	47 (8.6)	80 (14.2)
35-49 y	107 (26.3)	169 (19.2)	219 (24.4)	65 (24.9)	18 (25.9)	92 (22.3)	46 (31.8)	127 (21.9)	152 (26.2)	107 (21.6)	93 (20.2)
50-64 y	167 (26.5)	283 (34.0)	300 (25.7)	126 (35.9)	33 (35.1)	113 (28.7)	54 (26.4)	192 (26.7)	176 (27.9)	187 (28.7)	138 (31.4)
≥65 y	336 (30.4)	348 (31.4)	278 (28.4)	64 (21.2)	20 (14.0)	124 (24.2)	67 (25.5)	314 (32.3)	111 (18.3)	233 (34.9)	150 (27.8)
Health status and socioeconomic risk factors											
Multiple chronic conditions	250 (27.4)	248 (27.8)	108 (11.4)	41 (13.3)	15 (12.7)	85 (19.4)	40 (22.8)	188 (22.1)	63 (12.2)	138 (24.3)	168 (34.1)
Mental health condition	177 (27.6)	179 (27.2)	155 (17.5)	25 (6.7)	15 (23.9)	70 (19.4)	31 (19.7)	97 (16.4)	79 (13.6)	142 (28.3)	152 (26.3)
Experienced material hardship	58 (9.7)	82 (9.6)	102 (13.5)	20 (6.2)	5 (9.6)	24 (8.0)	2 (1.5)	26 (4.8)	77 (13.3)	40 (9.0)	74 (14.3)
Affordability of care											
Skipped needed medical care	121 (24.1)	104 (14.4)	102 (11.3)	32 (11.4)	7 (12.3)	63 (18.4)	9 (7.2)	45 (8.1)	115 (21.3)	31 (5.7)	191 (36.0)
Skipped dental care	175 (32.6)	190 (24.7)	180 (18.3)	46 (16.5)	9 (12.7)	142 (34.1)	24 (16.9)	113 (18.3)	123 (19.7)	75 (15.6)	179 (35.7)
Serious problems or unable to pay medical bills	45 (8.9)	47 (6.5)	85 (10.2)	12 (4.5)	5 (7.8)	25 (8.2)	6 (6.0)	36 (5.9)	44 (8.4)	13 (3.6)	113 (22.8)
Access to primary care											
Regular physician or place of care	644 (92.6)	837 (92.9)	952 (98.1)	283 (99.3)	83 (97.7)	407 (97.6)	186 (100.0)	654 (90.0)	539 (95.3)	611 (99.4)	461 (92.1)
Same day care needed last time	356 (54.4)	296 (34.9)	484 (52.2)	202 (74.7)	50 (71.0)	252 (60.0)	82 (50.9)	239 (36.7)	289 (56.6)	328 (56.2)	207 (47.3)
After-hours care difficult to access	394 (71.8)	502 (64.7)	419 (60.3)	127 (59.2)	16 (30.7)	147 (51.0)	38 (28.9)	254 (75.3)	193 (59.3)	291 (70.1)	241 (60.0)
Avoidable ED visit past 2 y	76 (31.4)	163 (38.4)	73 (25.5)	20 (33.3)	5 (38.0)	27 (21.1)	10 (34.6)	62 (31.2)	53 (39.2)	53 (29.2)	82 (51.9)

Abbreviation: ED, emergency department.

Regarding affordability of care indicators, among rural adults, the US had the highest rates for skipping needed medical (979 of 2489 [39.0%]) or dental (923 of 2489 [36.2%]) care and Norway and Sweden had the lowest (skipped medical care: Norway, 30 of 607 [8.2%]; Sweden, 229 of 2513 [11.2%]; skipped dental care: Norway, 86 of 607 [21.4%]; Sweden, 479 of 2513 [22.2%]). Norway and Sweden also reported the lowest rates of serious problems or inability to pay medical bills (Norway, 23 of 607 [6.3%]; Sweden, 157 of 2513 [7.5%]).

Participants living in rural areas with the least access to medical care lived in Canada, Sweden, and the US, as they had the lowest percentages of having a regular clinician or place of care (837 of 4530 [92.9%], 654 of 2513 [90.0%], and 461 of 2489 [92.1%], respectively), obtaining same-day care the last time it was needed (296 of 4530 [34.9%], 239 of 2513 [36.7%], and 207 of 2489 [47.3%], respectively), having difficulty accessing after-hours care (502 of 4530 [64.7%], 254 of 2513 [75.3%], and 241 of 2489 [60.0%], respectively), and the highest percentages of having an avoidable emergency department (ED) visit in the past 2 years (163 of 4530 [38.4%], 62 of 2513 [31.2%], and 82 of 2489 [51.9%], respectively). The country with the best access to medical care was the Netherlands, with 83 of 753 respondents (97.7%) having a regular clinician or place of care, and 50 (71.0%) obtaining same day care the last time needed. Only 16 (30.7%) had difficulty accessing after-hours care and 5 (38.0%) had an avoidable emergency department (ED) visit in the past 2 years.

The logistic regression results are shown in Table 3. Table 4 and the Figure contains the specific health indicators in which rural residence was either a protective or a risk factor, by country. We define rurality as protective if the rural vs urban odds ratio is less than 1 for health status and socioeconomic factors, and affordability care indicators and is greater than 1 for access to primary care indicators. Vice versa, rurality is considered a risk factor. Across the 11 countries and 10 health indicators, there were 21 occurrences of statistically significant rural vs urban health disparities; 13 of those where rural was a protective factor and 8 of those where rural was a risk factor. The mean number of geographic health disparities in the countries was 1.9, although there was a wide variation among the 11 nations (ranging between 0 to 5 disparities). The US had statistically significant geographic health disparities in 5 of the 10 indicators, the most of any country, followed by Switzerland (4), the UK and Australia (3 each), and France and Germany (2 each). Canada, Norway, and the Netherlands had no statistically significant geographic health disparities.

**Table 3.** Descriptive Statistics for Rural Within Indicator and Final Logistic Regression Model Differences for the Association of Rurality Between Countries Controlling for Age and Sex

Outcome	Rural participants, No. (%)		OR (95% CI)	Rural × country interaction	
	Yes	No		Wald χ^2^	*P* value
**Health status and socioeconomic factors**					
Multiple chronic conditions					
Australia (n = 2134)	250 (38.0)	429 (28.4)	1.02 (0.75-1.40)	0.72	.70
Canada (n = 5084)	248 (20.8)	646 (14.7)	1.02 (0.76-1.38)		
France (n = 3026)	108 (33.9)	866 (34.3)	0.86 (0.62-1.20)		
Germany (n = 1001)	41 (23.5)	243 (28.7)	0.75 (0.48-1.17)		
Netherlands (n = 753)	15 (12.8)	69 (11.7)	1.34 (0.67-2.67)		
New Zealand (n = 1003)	85 (52.6)	336 (43.5)	1.10 (0.67-1.80)		
Norway (n = 607)	40 (40.3)	146 (32.8)	1.21 (0.66-2.20)		
Sweden (n = 2506)	188 (28.9)	523 (27.5)	0.81 (0.64-1.02)		
Switzerland (n = 2283)	63 (23.9)	498 (27.8)	0.92 (0.63-1.32)		
UK (n = 1990)	138 (28.9)	478 (21.3)	1.02 (0.70-1.50)		
US (n = 2483)	168 (22.1)	328 (17.0)	1.04 (0.92-1.19)		
Mental health conditions					
Australia (n = 2129)	177 (30.6)	502 (30.7)	1.10 (0.79-1.52)	2.15	.02
Canada (n = 5065)	179 (15.8)	712 (16.3)	1.13 (0.84-1.51)		
France (n = 3021)	155 (39.5)	819 (33.3)	1.32 (1.00-1.74)		
Germany (n = 1000)	25 (18.2)	259 (29.0)	0.50 (0.29-0.87)		
Netherlands (n = 750)	15 (18.7)	69 (10.7)	1.90 (0.98-3.69)		
New Zealand (n = 998)	70 (43.3)	350 (45.4)	0.92 (0.59-1.42)		
Norway (n = 605)	31 (33.8)	154 (34.1)	0.99 (0.56-1.74)		
Sweden (n = 2487)	97 (24.4)	603 (28.2)	0.87 (0.66-1.13)		
Switzerland (n = 2280)	79 (24.2)	482 (27.9)	0.82 (0.58-1.15)		
UK (n = 1983)	142 (24.4)	471 (22.1)	1.27 (0.91-1.79)		
US (n = 2481)	152 (16.7)	342 (19.1)	0.89 (0.80-0.99)		
Experiences material hardship					
Australia (n = 2133)	58 (31.6)	621 (30.4)	1.25 (0.74-2.09)	1.00	.44
Canada (n = 5085)	82 (12.0)	812 (16.5)	0.81 (0.54-1.23)		
France (n = 3022)	102 (35.4)	873 (34.2)	1.12 (0.82-1.53)		
Germany (n = 1002)	20 (28.0)	264 (27.8)	1.00 (0.54-1.84)		
Netherlands (n = 751)	5 (21.9)	79 (11.3)	2.09 (0.74-5.95)		
New Zealand (n = 1003)	24 (41.4)	397 (45.4)	0.95 (0.50-1.82)		
Norway (n = 607)	2 (17.6)	184 (34.8)	0.42 (0.06-3.06)		
Sweden (n = 2508)	26 (20.9)	686 (28.3)	0.74 (0.45-1.20)		
Switzerland (n = 2280)	77 (22.1)	483 (28.3)	0.69 (0.49-0.97)		
UK (n = 1989)	40 (18.7)	575 (23.2)	0.92 (0.53-1.58)		
US (n = 2488)	74 (16.2)	422 (18.9)	0.87 (0.77-0.99)		
**Affordability of care**					
Skipped needed medical care					
Australia (n = 2131)	121 (34.1)	558 (29.6)	1.52 (1.06-2.18)	1.94	.04
Canada (n = 5079)	104 (16.0)	789 (16.0)	1.20 (0.82-1.75)		
France (n = 3024)	102 (34.9)	873 (34.2)	1.10 (0.79-1.51)		
Germany (n = 1003)	32 (30.3)	253 (27.7)	1.16 (0.70-1.91)		
Netherlands (n = 749)	7 (15.6)	77 (11.5)	1.38 (0.59-3.25)		
New Zealand (n = 998)	63 (46.4)	356 (44.5)	1.21 (0.76-1.93)		
Norway (n = 607)	9 (30.0)	177 (34.7)	0.87 (0.33-2.26)		
Sweden (n = 2422)	45 (19.9)	639 (28.4)	0.70 (0.49-1.02)		
Switzerland (n = 2103)	115 (22.7)	402 (28.5)	0.72 (0.53-0.96)		
UK (n = 1976)	31 (13.5)	580 (23.8)	0.61 (0.34-1.11)		
US (n = 2446)	191 (17.1)	300 (19.5)	0.94 (0.84-1.05)		
Skipped dental care					
Australia (n = 2135)	175 (31.5)	504 (30.1)	1.27 (0.92-1.74)	2.55	<.001
Canada (n = 5089)	190 (14.5)	704 (16.5)	0.97 (0.73-1.30)		
France (n = 3028)	180 (33.8)	796 (34.4)	1.02 (0.79-1.33)		
Germany (n = 1004)	46 (23.9)	239 (28.9)	0.74 (0.48-1.14)		
Netherlands (n = 753)	9 (14.7)	75 (11.5)	1.29 (0.57-2.93)		
New Zealand (n = 1003)	142 (41.4)	279 (47.1)	0.88 (0.62-1.25)		
Norway (n = 607)	24 (27.0)	162 (36.3)	0.68 (0.37-1.25)		
Sweden (n = 2513)	113 (22.9)	599 (29.1)	0.78 (0.60-1.01)		
Switzerland (n = 2284)	123 (20.3)	438 (29.8)	0.58 (0.44-0.78)		
UK (n = 1991)	75 (17.1)	541 (24.2)	0.70 (0.47-1.05)		
US (n = 2488)	179 (18.2)	317 (18.5)	1.05 (0.94-1.16)		
Serious problems or unable to pay medical bills					
Australia (n = 2135)	45 (28.9)	634 (30.7)	1.05 (0.60-1.82)	0.37	.96
Canada (n = 5089)	47 (14.6)	847 (16.1)	1.03 (0.59-1.80)		
France (n = 3028)	85 (34.6)	891 (34.2)	1.06 (0.75-1.51)		
Germany (n = 1004)	12 (30.6)	273 (27.8)	1.13 (0.52-2.44)		
Netherlands (n = 753)	5 (17.1)	79 (11.6)	1.53 (0.51-4.56)		
New Zealand (n = 1003)	25 (47.7)	396 (44.8)	1.20 (0.59-2.45)		
Norway (n = 607)	6 (32.4)	180 (34.4)	0.94 (0.33-2.67)		
Sweden (n = 2513)	36 (21.8)	676 (28.2)	0.77 (0.51-1.17)		
Switzerland (n = 2284)	44 (25.6)	517 (27.5)	0.89 (0.58-1.36)		
UK (n = 1991)	13 (21.0)	603 (22.8)	1.05 (0.48-2.31)		
US (n = 2488)	113 (18.7)	383 (18.3)	1.09 (0.97-1.22)		
**Access to primary care**					
Regular physician or place of care					
Australia (n = 2135)	644 (30.2)	35 (34.6)	0.58 (0.31-1.06)	4.11	<.001
Canada (n = 5089)	837 (16.8)	57 (11.3)	1.10 (0.68-1.76)		
France (n = 3028)	952 (35.2)	24 (14.0)	2.96 (1.80-4.86)		
Germany (n = 1004)	283 (28.9)	2 (5.0)	6.98 (1.41-34.50)		
Netherlands (n = 753)	83 (11.7)	1 (32.5)	0.30 (0.03-2.92)		
New Zealand (n = 1003)	407 (45.6)	14 (28.7)	1.62 (0.78-3.39)		
Norway (n = 607)	186 (34.3)	0 (0.00)	0.82 (0.62-1.10)		
Sweden (n = 2513)	654 (28.8)	58 (20.8)	1.29 (0.91-1.82)		
Switzerland (n = 2284)	539 (27.9)	22 (18.6)	1.91 (1.08-3.36)		
UK (n = 1991)	611 (23.7)	5 (4.0)	5.29 (1.62-17.25)		
US (n = 2488)	461 (19.0)	35 (13.5)	1.32 (1.10-1.58)		
Same day care needed last time					
Australia (n = 2073)	307 (25.4)	356 (39.6)	0.54 (0.40-0.73)	3.15	<.001
Canada (n = 4685)	296 (14.4)	519 (16.5)	0.88 (0.68-1.16)		
France (n = 2829)	484 (34.2)	445 (35.8)	0.94 (0.77-1.15)		
Germany (n = 970)	202 (28.3)	77 (28.1)	1.02 (0.73-1.45)		
Netherlands (n = 693)	50 (12.5)	27 (10.1)	1.28 (0.73-2.23)		
New Zealand (n = 954)	252 (45.4)	155 (46.5)	1.01 (0.71-1.43)		
Norway (n = 572)	82 (36.7)	90 (31.3)	1.29 (0.83-2.02)		
Sweden (n = 2246)	239 (31.7)	408 (26.9)	1.30 (1.05-1.61)		
Switzerland (n = 2137)	289 (29.3)	246 (25.7)	1.20 (0.94-1.53)		
UK (n = 1930)	328 (25.3)	279 (21.2)	1.35 (1.00-1.82)		
US (n = 2134)	207 (17.5)	222 (18.9)	0.96 (0.86-1.07)		
After-hours care difficult to access					
Australia (n = 1711)	394 (49.5)	156 (15.5)	5.16 (3.73-7.13)	8.68	<.001
Canada (n = 4339)	502 (17.0)	261 (13.9)	1.20 (0.92-1.56)		
France (n = 2170)	419 (36.7)	291 (31.5)	1.26 (1.00-1.59)		
Germany (n = 658)	127 (33.5)	78 (26.2)	1.35 (0.94-1.95)		
Netherlands (n = 445)	16 (15.3)	41 (13.5)	1.16 (0.57-2.36)		
New Zealand (n = 769)	147 (50.1)	165 (37.9)	1.63 (1.10-2.40)		
Norway (n = 416)	38 (30.7)	98 (41.5)	0.61 (0.35-1.06)		
Sweden (n = 1188)	254 (27.5)	81 (29.0)	0.92 (0.66-1.27)		
Switzerland (n = 1286)	193 (26.2)	125 (27.0)	0.96 (0.70-1.32)		
UK (n = 1477)	291 (24.9)	167 (18.3)	1.44 (1.02-2.02)		
US (n = 2017)	241 (21.2)	169 (16.6)	1.29 (1.17-1.43)		
Avoidable ED visit past 2 y					
Australia (n = 606)	76 (39.3)	159 (37.7)	1.22 (0.72-2.06)	3.61	<.001
Canada (n = 1868)	163 (18.8)	212 (19.1)	1.09 (0.74-1.62)		
France (n = 810)	73 (37.6)	197 (36.0)	1.11 (0.72-1.72)		
Germany (n = 265)	20 (32.1)	49 (25.3)	1.41 (0.70-2.86)		
Netherlands (n = 141)	5 (13.5)	9 (10.1)	1.28 (0.33-5.01)		
New Zealand (n = 235)	27 (41.3)	89 (54.9)	0.67 (0.30-1.47)		
Norway (n = 134)	10 (37.2)	25 (27.4)	1.51 (0.52-4.40)		
Sweden (n = 776)	62 (30.8)	154 (26.3)	1.35 (0.91-2.02)		
Switzerland (n = 618)	53 (30.8)	99 (27.0)	1.20 (0.75-1.92)		
UK (n = 634)	53 (22.2)	141 (24.4)	1.02 (0.57-1.83)		
US (n = 745)	82 (28.1)	91 (16.5)	2.15 (1.90-2.44)		

**Table 4.** Comparison of Statistically Significant Rural and Urban Outcomes by Country

Outcome	Better in rural	Better in urban
Has mental health conditions	Germany, US	France
Experiences material hardship	Switzerland, US	Australia
Skipped needed medical care because of cost	Switzerland	Australia
Skipped needed dental care because of cost	Switzerland	Australia, New Zealand, UK, US
Has a regular physician or place of care	Germany, France, Switzerland, UK, US	US
Same day care last time needed	Sweden, UK	NA

Abbreviation: NA, not applicable.

**Figure.**
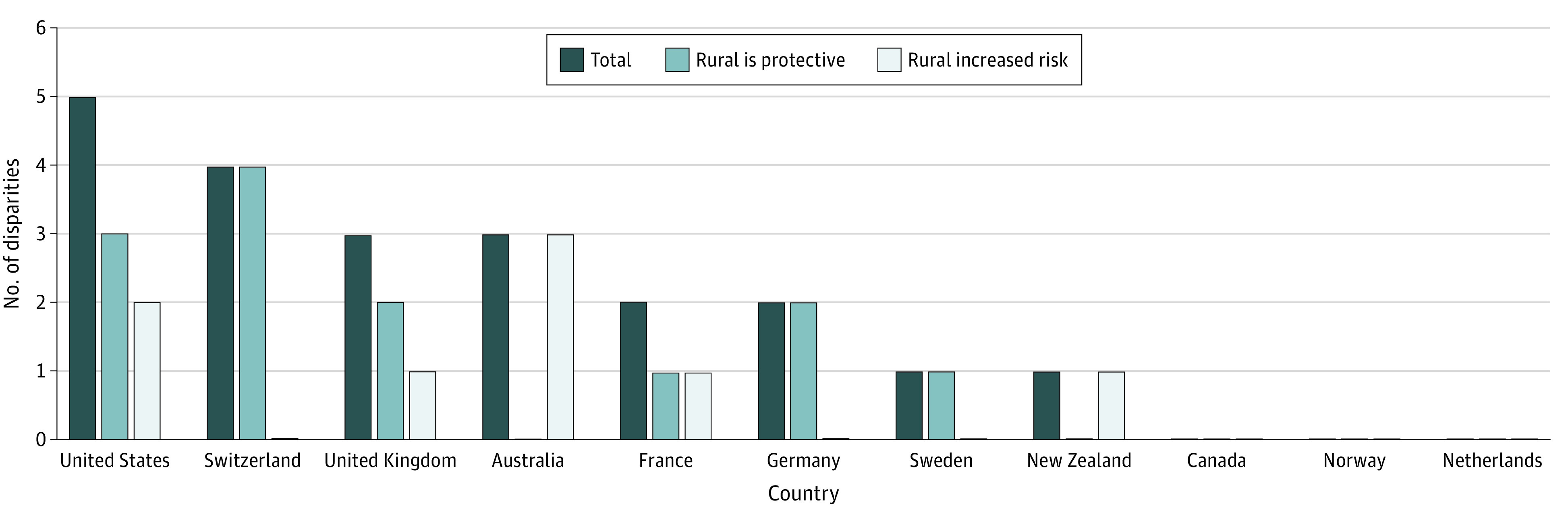
Geographic Health Disparities in 11 High-Income Nations

Table 4 also reveals the variation in the number of geographic health disparities across the 10 indicators and 3 domains. For example, we found no occurrence of geographic health disparities for 2 of the indicators, whereas 5 nations had a geographic health disparity for 1 indicator. The indicators with the most occurrences of geographic health disparities were in the access to care domain. Rural setting was associated with increased odds of having a regular clinician or place of care for Germany, France, Switzerland, the UK, and the US, and was associated with decreased odds of accessing after-hours care in Australia, New Zealand, the UK, and the US. The indicators with no occurrence of disparities were having multiple chronic conditions and being unable to pay medical bills.

## Discussion

This study used data from the 2020 Commonwealth Fund IHP Survey to compare differences in the association of indicators of health status and socioeconomic risk, affordability of care, and access to care, with geographic area type (rural vs urban) between 11 high-income countries.

The US had the highest number of geographic health disparities among the 11 nations. This finding is consistent with past analyses showing that the US ranks lowest out of other high-income countries on health outcomes and equity.^31,33^ Canada, Norway, and the Netherlands were the only countries with no urban-rural disparities. It is unclear why these countries did not have any geographic health disparities. Past studies of geographic health disparities in Canada found that living in a rural area was a risk factor for obesity^20^ (but not for depression^34^), and in Norway, municipalities with low population density were associated with higher mortality rates.^35^ From these countries, further research is warranted to determine what underlying factors contribute to geographic health disparities and if policies and practices from them can be adopted elsewhere to improve health equity.

Among the 10 indicators and 3 domains, the broad variation in countries with geographic health disparities elucidated more questions than answers. In our analysis, there were no occurrences of disparities for having serious problems or being unable to pay medical bills and having multiple chronic conditions, yet previous studies have demonstrated rural residents have lower socioeconomic status^36,37^ and higher rates of multiple chronic conditions.^3,37,38^ It was also perplexing why there were not more instances of geographic health disparities, both within the access to care domain and overall. Health system differences significantly affect these results (systemic differences across the countries available in eTable 2 in Supplement 1). Besides health system differences, the systematic cross-country variation in the interpretation of questions, unobserved characteristics, and events that might have influenced responses^39^ could be the reasons behind these results. However, it was not surprising that most disparities were found in the access to care domain.

Another unexpected result was that rurality was found to be protective more often than a risk factor when looking at all the countries and health indicators cumulatively. One of these factors could be attributed to a plausible underdiagnoses of mental health in rural areas that might make it look like there a lower rate of mental illness in rural areas. As noted above, the systematic differences in the interpretation of survey questions^39^ and cultural factors such as stoicism^40^ and stigma^41^ could be driving these results. Notably, rural dwellers had greater odds of having a regular clinician or place of care more often than urban dwellers in Germany, France, Switzerland, the UK, and US. This was surprising, given the shortage of health care professionals in rural areas.^42^

One European country, Switzerland, stood out for having 4 occurrences of geographic health disparities, more than any other country besides the US. This is surprising, given that Switzerland has the second highest life expectancy in Europe, spends more per capita than any European country, and has high consumer satisfaction.^43^ However, a 2021 study of preventable hospitalizations in Switzerland found high regional variability, a finding consistent with past research,^44^ but it did not describe the size or other characteristics of the regions.^45^

In summary, the US should look to Canada, Norway, and the Netherlands for policies to improve geographic health equity. Future studies should explore new methods of defining and operationalizing rurality to increase the accuracy and validity of cross-country comparison.

### Strengths and Limitations

This study had several strengths. It is the first study, as far as we know, to compare geographic health disparities across a large group of high-income countries. The results show that indicators of socioeconomic and health status, affordability of care, and access to care vary widely across the 11 countries and that geographic health disparities are relatively common.

This study also had several limitations, the main one being the lack of consistent or standardized definition for rurality. As has been noted elsewhere, there are conflicting and inconsistent definitions of rural and urban both globally^46^ and within the US.^47,48^ Unlike other countries included in the study, the geographic disparities in the US have a background of racially discriminating policies. Studies suggest that these discriminatory policies have adversely affected health outcomes for racial and ethnic minority populations.^49,50,51,52,53,54,55^ Our study does not delve into such country-specific structural determinants of geographic disparities.

Due to data limitations, we could not control for race and ethnicity or education, which could lead to possible confounding factors. Also, a limited response rate could be a possible threat to the analysis. However, the response rate of 14% to 49% is not considered a severe limitation due to the population weighting in random-digit-dialing telephone surveys, a technique that research has shown can minimize selection bias and is commensurate with other studies.^56^ As with all cross-sectional surveys, data was collected at a single time point and could have been influenced by world or national events in each of the countries. Self-reporting could have also biased the results. Our results should be interpreted keeping these limitations in mind.

## Conclusion

In this survey study of 11 high-income nations, health disparities across 10 indicators were identified. Health policy makers in the US should look to Canada, Norway, and the Netherlands to improve geographic-based health equity.

## Notes

Supplement 1. **eTable 1.** Rurality Definition for Each Country **eTable 2.** Health Care System Performance Ranking 2021 and Health Insurance Coverage

Supplement 2.
Data Sharing Statement
