# Supplementary material for: Mapping Health Disparities in 11 High-Income Nations
Source: JAMA Netw Open. 2023 Jul 7;6(7):e2322310. doi: 10.1001/jamanetworkopen.2023.22310 (PMC10329207; doi:10.1001/jamanetworkopen.2023.22310)
Supplement: Supplement 1. — eTable 1. Rurality Definition for Each Country eTable 2. Health Care System Performance Ranking 2021 and Health Insurance Coverage [file jamanetwopen-e2322310-s001.pdf]

Supplemental Online Content

MacKinnon NJ, Emery V, Waller J, et al. Mapping health disparities in 11 high-income nations. *JAMA Netw Open.* 2023;6(7):e2322310. doi:10.1001/jamanetworkopen.2023.22310

eTable 1. Rurality Definition for Each Country

eTable 2. Health Care System Performance Ranking 2021 and Health Insurance Coverage

This supplemental material has been provided by the authors to give readers additional information about their work.

© 2023 MacKinnon NJ et al. *JAMA Network Open.*

**eTable 1.** Rurality Definition for Each Country	
Country	Definition
Australia	Postal Code variable in the IHP Survey (DZA) combined with definition of rural /urban postal codes at https://www.agriculture.gov.au/biosecurity-trade/import/online-services/delivery-postcode-classifications
Canada	Question in the IHP Survey Community Size (Q620). Rural was defined as <5000 or 5000-99,999.
France	Postal Code variable in the IHP Survey (D-ZF) combined with National Institute of Statistics and Economic Studies (INSEE.fr) postal code, population density, and rurality definitions. Rural was defined as "Sparsely Populated Commune" and "Very Sparsely Populated Commune."
Germany	Question in the IHP Survey (Q2150): "Which of the following describes where you live?" Rurality was defined as "Village or rural location."
The Netherlands	Regions/Provinces question in the IHP Survey (Q630) and province-based population density from Statistics Netherlands CBS Open data StatLine. The population density of the region from StatLine and the World Bank definition of rurality based on population density of "Rural areas (thinly populated areas) <100 inhabitants/km^2" was used to define rurality.
New Zealand	Question in the IHP Survey (DNZU): "Would you say your living area is…" and defined rurality as "A regional or rural area."
Norway	Question in the IHP Survey (Q2150): "Which of the following describes where you live?" Rurality was defined as "Village or rural location."
Sweden	Question in the IHP Survey Community Type (Q617) and defined rurality as the World Bank definition of rurality based on population density of "Rural areas (thinly populated areas) <100 inhabitants/km.^{2}"
Switzerland	Question in the IHP Survey Community Type (Q615). Rurality was defined as a "Village or rural Location".
United Kingdom	Question in the IHP Survey (Q2150): "Which of the following describes where you live?" Rurality was defined as "Village or rural location".
United States	Used the zip code variable DZ1 f and the https://www.hrsa.gov/rural-health/about-us/what-is-rural/data-files that gives those zip codes which are in rural areas.

© 2023 MacKinnon NJ et al. *JAMA Network Open.*

e Table 2: Health Care System Performance Ranking 2021 and Health Insurance Coverage[a]

	AUS	CAN	FRA	GER	NETH	NZ	NOR	SWE	SWI	UK	US
**Overall Ranking[b]**	**3**	**10**	**8**	**5**	**2**	**6**	**1**	**7**	**9**	**4**	**11**
Access to Care	8	9	7	3	1	5	2	6	10	4	11
Care Process	6	4	10	9	3	1	8	11	7	5	2
Administrative Efficiency	2	7	6	9	8	3	1	5	10	4	11
Equity	1	10	7	2	5	9	8	6	3	4	11
Health Care Outcomes	1	10	6	7	4	8	2	5	3	9	11
**Health Insurance Coverage (%)[c]**											
Public	100	100	100	88	100	100	100	100	100	100	34
Private	46-55	67	95	10.6	84	33	10	6	-	10.5	67
**Maximum out-of-pocket costs per year (safety net)[c,d]**											
	322	N/A	63	N/A	493	7-17	281	120	2645	N/A	Variable

Abbreviation: N/A, Not Applicable

Footnotes:

a: Prior permission from Commonwealth Fund was sought for the data reproduction.

b: Schneider, E.C.; Shah, A.; Doty, M.M.; Tikkanen, R.; Fields, K.; Williams, R.; II, M.M. Reflecting Poorly: Health Care in the US Compared to Other High-Income Countries. *New York: The Commonwealth Fund* **2021**.

c: Tikkanen, R.; Osborn, R.; Mossialos, E.; Djordjevic, A.; Wharton, G. International profiles of health care systems. *The Commonwealth Fund* **2020**.

d: Shown in US dollars for primary care visits for an adult.

© 2023 MacKinnon NJ et al. *JAMA Network Open.*
