# Supplementary material for: Mapping Health Disparities in 11 High-Income Nations
Source: JAMA Netw Open. 2023 Jul 7;6(7):e2322310. doi: 10.1001/jamanetworkopen.2023.22310 (PMC10329207; doi:10.1001/jamanetworkopen.2023.22310)
Supplement: Supplement 2. — Data Sharing Statement [file jamanetwopen-e2322310-s002.pdf]

Data Sharing Statement

MacKinnon. Mapping Health Disparities in 11 High-Income Nations. *JAMA Netw Open*.
Published July 07, 2023. doi:10.1001/jamanetworkopen.2023.22310

Data
Data available: No

Additional Information
Explanation for why data not available: The data is owned by the Commonwealth Fund.
